# Shifting focus to adolescent wellbeing and inclusive participation in the digital age

**DOI:** 10.5694/mja2.52653

**Published:** 2025-04-24

**Authors:** Stephanie R Partridge, Allyson R Todd, Si Si Jia, Rebecca Raeside, with the Health Advisory Panel for Youth at the University of Sydney (HAPYUS), Sara Wardak, Shuwei Guo, Yi Ying Lim, Moudasir Jalili, Lucy Goodyer, Lucy Gee, Lucy Agland, Natalie Ryan, Nitika Sharma, Caitlyn Lee, Chloe Caldwell, Elena Wang

**Affiliations:** ^1^ University of Sydney Sydney NSW; ^2^ Charles Perkins Centre University of Sydney Sydney NSW

**Keywords:** Adolescent medicine, Consumer health information, Chronic disease, Prevention and control, eHealth

Older generations often say the “youth of today aren't resilient” yet Gen Z faces a rapidly changing world, such as COVID‐19, climate change and technological advancements.

HAPYUS Cohort 2024

## Positionality statement

This perspective article is co‐authored by a diverse team of researchers and youth advisors. The researchers all identify as women and bring interdisciplinary expertise in adolescent health, nutrition, digital health, public health, and youth engagement. Collectively, we have held leadership roles in the International Association for Adolescent Health, Australian Association for Adolescent Health, and the Public Health Association of Australia. Our research is supported by funding from the Medical Research Future Fund, New South Wales Health, and the National Heart Foundation of Australia. The youth advisory group comprises adolescents aged 14–18 years from diverse cultural, geographic, and socio‐economic backgrounds across NSW. Specifically, in the second cohort, 50% are aged 14–16 years, 50% are aged 17–18 years, 75% identify as girls/women, 44% speak a language other than English at home, and 25% live in rural or regional NSW. Young people in the Health Advisory Panel for Youth at the University of Sydney (HAPYUS) independently applied to join, submitting written statements that demonstrated significant maturity, commitment, and a strong passion for creating healthier societies for their peers. Their lived experience growing up in a digital era has directly shaped the perspectives presented in this article, with the research team supporting the integration of evidence. These youth are not participants but collaborators, and their ability to contribute meaningfully, combined with increasing recognition of adolescents’ capacity to provide informed consent from age 14, supports their inclusion as co‐authors without requiring parental or guardian consent.

## Introduction

Australia is home to 4.6 million adolescents, aged ten to 24 years, which is over 18% of the total population.^1^ Our society is not conducive to optimising their wellbeing, despite evidence of associations between wellbeing and chronic disease outcomes and risks in adulthood, such as cardiovascular disease.^2^ Australia's growing burden of chronic diseases necessitates re‐evaluating our current approaches, encouraging all sectors to collaborate with adolescents on health and wellbeing initiatives.^3^

## The importance of engaging with young people

Engaging adolescents in designing digital health initiatives is crucial for promoting wellbeing.^4^ Genuine partnerships that incorporate adolescents’ creativity, ideas and concerns can enhance the quality of digital health solutions.^5^ This aligns with the Convention on the Rights of the Child, which Australia ratified in 1990.^6^ When adolescents actively create solutions that affect them, they are more likely to be effective and resonate with their peers.^7^

Despite progress, youth voices remain under‐represented, with less than 1% of all empirical adolescent health research including a youth voice.^8^ Recent developments, such as the federal government Office for Youth and the Engage! strategy, signal a shift toward valuing adolescent participation in decision making.^9^ However, research and government institutions remain largely adult‐centred. Greater emphasis on inclusive dialogue, power sharing, and co‐design is needed to ensure adolescents are supported to contribute meaningfully.^10^, ^11^, ^12^ The National Preventive Health Strategy,^13^ the Engage! strategy^9^ and the National Digital Health Strategy^14^ advocate for participatory processes and digitally enabled health services. Together, these initiatives highlight a growing commitment to holistic, inclusive, and digital‐first approaches to adolescent wellbeing.

## Digital determinants of health for digital natives

Adolescents are more digitally connected than any other generation, with mobile coverage reaching 99% of our population and near‐ubiquitous smartphone access. Increased reliance on digital technologies perpetuated during the lockdowns related to the coronavirus disease 2019 (COVID‐19) pandemic for education, social connection and government communication. Yet, access varies substantially across Australia in rural areas and among different culturally and linguistically diverse groups, which may lead to a digital divide. Nevertheless, digitally enabled initiatives offer immense untapped opportunities to optimise the wellbeing of adolescents using their participation and creativity in developing solutions.^15^ Attention must be paid to addressing the digital determinants of health to achieve positive wellbeing outcomes. These determinants are technological factors that need to be considered when developing programs such as affordability, accessibility and quality of services.^16^ Even though digital technology is a double‐edged sword with many well established negative impacts, it is ingrained in our society and everyday life. We must overcome the challenges to maximise the potential of digital technologies to enhance adolescent wellbeing.

## Conceptualising adolescent wellbeing in the digital age

Wellbeing is defined as adolescents having the support, confidence and resources to thrive in secure and healthy relationships, realising their full potential and rights.^17^ The adolescent wellbeing framework provides a holistic construct of wellbeing covering five interconnected domains: (i) good health and optimum nutrition; (ii) connectedness, positive values and contribution to society; (iii) safety and a supportive environment; (iv) learning, competence, education, skills and employability; and (v) agency and resilience.^17^

The authorship team includes researchers and youth advisory group members, aged between 14 and 18 years, with diverse backgrounds and experiences. Full details about the youth advisory group structure and processes are available elsewhere as part of a formal evaluation of the first cohort.^18^ In addition, the group's structure aligns with the recommendations outlined in the guidelines for the design and implementation of youth participation initiatives to safeguard mental health and wellbeing.^19^

The authors explored the five adolescent wellbeing domains in the context of digital health, defined as development, use or regulation of digital technologies to improve health and wellbeing. To facilitate meaningful youth participation, we employed deliberate dialogue, which is a structured process of discussion and reflection that enables a group to consider diverse perspectives and collaboratively work towards consensus. Adult researchers integrated supporting referenced evidence. This method was chosen to address power imbalances within the authorship team, ensuring that young people's perspectives were meaningfully integrated. Discussions were conducted through group sessions, with all authors contributing to and approving the ideas expressed here. This approach stresses the importance of youth co‐authorship on perspectives that are directly relevant to them.

## Domain 1: good health and optimum nutrition

Digital health interventions can have an important role in promoting good health and optimum nutrition among adolescents.^20^ However, there is a need to improve their critical health literacy.^21^ Adolescents want to confidently evaluate the health information they find online, including social media. Co‐designed educational interventions involving adolescents and health providers are necessary to achieve critical decision‐making skills.^21^ For example, the Health4Me intervention is a universal program that aims to improve adolescents’ health and nutrition. The intervention was co‐designed with adolescents and is delivered via text messages eliminating access barriers (eg, mobile data).^22^, ^23^ This intervention is currently being tested in a randomised controlled trial, with outcomes available in 2025.

Access to reliable online information persists. For example, there are limited engaging and interactive websites with health information to improve health and nutrition for adolescents.^24^ Very few websites are explicitly written for adolescents (most are targeted at parents); none are of excellent quality and they do not use easy to understand language. In addition, adolescents have raised concerns about misinformation and weight stigma online, particularly from social media influencers. Some progress was made in 2019, when Instagram restricted the promotion of certain weight‐loss products from users known to be aged under 18 years, and any claim of “miraculous” weight loss linked to commercial offers was banned from the site. In 2022, the Australian Therapeutic Goods Administration restricted how influencers can promote regulated products, including supplements and protein powders. However, physique‐focused nutrition‐related content, primarily by influencers, remains popular on social media, increasing adolescents’ exposure to harmful misinformation and weight stigma.^25^, ^26^

Addressing these concerns will require governments to implement further regulations regarding the quality of health information on social media platforms and provide education on critical health literacy skills. The cumulative effect of interconnected strategies will expose adolescents to reliable, credible and strengths‐based information and content aligned with national guidelines, and recommendations for good health and nutrition during adolescence.

## Domain 2: connectedness, positive values and contribution to society

Digital technologies such as social media platforms can foster connectedness among adolescents, helping them maintain relationships and build social networks. Adolescents also express concerns about peer pressure and loneliness. Effective digital technologies should address these dual aspects, promoting positive connections while mitigating negative impacts.

Moreover, digital technologies can promote positive values and encourage adolescents to contribute to society. Social media platforms provide opportunities for adolescents to identify and pursue interests, build identity, and engage in community support networks. Highlighting these positive contributions can inspire more adolescents to use digital technologies for societal benefit. Examples include successful digital initiatives such as UNICEF Australia Young Ambassadors^27^ and our youth advisory group, the Health Advisory Panel for Youth at the University of Sydney (HAPYUS), which fosters connections between diverse adolescents, researchers and the science community.^28^ Without the broad reach of social media, engaging a broad population of adolescents would be challenging, limiting opportunities to known networks and potentially excluding many young people, thereby disregarding their opinions and voices.

Although there is consensus on limiting adolescents’ exposure to current social media environments that foster negative experiences — Australia has banned people aged under 16 years from using social media — this can be seen as a temporary solution that does not address the root causes of such negative experiences. Over four in ten teens had at least one negative online experience in the six months to September 2020, increasing to over 50% of those aged 14–17 years.^29^ More accountability from social media companies is needed. According to the Engage! strategy, nearly 40% of adolescents want to engage with government decision making via social media.^9^ When asked how they would like to hear about government actions, nearly 70% of adolescents preferred social media. Therefore, restricting access for those aged 12–16 years might limit their engagement and voice in societal matters. New digital communication strategies that are inclusive and wide‐reaching will need greater investment.

## Domain 3: safety and a supportive environment

Creating safe digital environments is essential to protect adolescents from cyberbullying, privacy breaches, and harmful content, while enabling meaningful social connections. Adolescents report negative online experiences, such as exposure to harmful content, desensitisation, and the normalisation of discrimination. Addressing these concerns requires stronger content regulations, algorithm transparency, and youth‐led awareness platforms.

Adolescents’ everyday engagement with digital technologies increases their digital footprint, raising concerns about privacy, safety and future employability. Many young people remain unaware of how their data are stored or used, particularly with artificial intelligence (AI) platforms (such as ChatGPT and Meta AI) and social media interactions.

Australia's eSafety Commissioner provides education and removes harmful content, including cyberbullying.^29^ However, gaps remain in regulating digital marketing of unhealthy food and beverages to adolescents. By age 13 years, advertisers collect over 72 million data points per child, shaping their interests and behaviours.^30^ There was strong support among leading public health and medical organisations to limit unhealthy food marketing to Australian adolescents in a recent public consultation. Calls for strong government‐led policy suggest mandatory regulation to ensure compliance does not rely upon voluntary commitment or self‐regulation of harmful industries, eliminating companies’ potential for market gain through non‐compliance.

## Domain 4: learning, competence, education, skills and employability

Digital technologies can enhance learning and skill development, preparing adolescents for future employment. However, digital equity issues in learning and education remain, with variability in internet access, affordability, accessibility and digital literacy posing substantial challenges. Ensuring all adolescents have access to digital resources is crucial for their development. Promoting diverse learning platforms, updating education curriculums with youth engagement, and supporting adolescents seeking employment are essential steps.

Recent research has highlighted concerns about the equity of digital technologies for health and wellbeing promotion.^31^ Issues were raised about the digital literacy skills needed to engage in these programs, particularly for adolescents most in need. Digital literacy is rarely measured or considered in digital health research, yet adolescents want to enhance these skills. Embedding co‐designed critical health literacy programs as part of digital citizenship programs within education settings will benefit adolescents now and equip them with key skills for future education and employment.

## Domain 5: agency and resilience

Empowering adolescents to control their digital footprint will help foster agency and resilience. Digital technologies that are appropriately regulated, adequately consider data privacy, and are youth‐centred are essential. Implementing robust data protection policies and clear guidelines on data usage builds trust and provides a sense of security.

Developing digital resilience can be enhanced by supporting adolescents in engaging in diverse digital communities with appropriate safeguards. This can only be achieved through greater government commitment to regulate the private technology sector better and by providing education on digital literacy and critical thinking, helping adolescents make informed decisions.^32^ Incorporating digital citizenship programs in schools can teach responsible online behaviour, cyber ethics, and the long term implications of digital actions, promoting a positive digital identity.

Involving adolescents in co‐designing digital health tools such as Health4Me^23^ ensures a youth‐centred program that provides users with a relevant and acceptable experience, leading to increased engagement and ownership. Empowering adolescents to have control over digital health technologies has the potential to enhance their sense of agency through collective actions that contribute to improving the wellbeing of their generation.

## Conclusions and recommendations

Governance of digital technologies has fallen behind the pace of technological innovation and the evidence base on how digitalisation can support or harm adolescent wellbeing. A holistic approach to digital health that promotes adolescent wellbeing across all domains is essential. Specific policy changes and multisectoral collaboration are needed to address the digital determinants of health in Australia. Researchers, health professionals and governments should work in direct partnership with adolescents, focus on creating safe, supportive, and equitable digital environments. This approach ensures that adolescents are not just passive recipients of digital health tools but active contributors to their development. Further, these tools are more likely to be adopted, implemented and serve adolescents’ needs to help them thrive in the digital age.

### Ethical considerations

Ethics approval was not required for youth advisors to co‐author this perspective article, as no personal health or demographic data were collected or reported. This approach was supported by advice from the University of Sydney Human Research Ethics Committee and aligns with the National Health and Medical Research Council Statement on Consumer and Community Involvement in Health and Medical Research.

## Open access

Open access publishing facilitated by The University of Sydney, as part of the Wiley – The University of Sydney agreement via the Council of Australian University Librarians.

## Competing interests

No relevant disclosures.

## Provenance

Commissioned; externally peer reviewed.
